# Voice problems in chronic cough: prevalence and implications for health and sick leave in a Northern European population

**DOI:** 10.1186/s12890-025-03877-6

**Published:** 2025-08-21

**Authors:** Sofia Ögefeldt, Greta Öhlund Wistbacka, Ellika Schalling, Henrik Johansson, Andrei Malinovschi, Christer Janson, Lars Modig, Mathias Holm, Rain Jögi, Christine Cramer, Thorarinn Gislason, Ane Johannessen, Össur Ingi Emilsson

**Affiliations:** 1 Department of Public Health and Caring Sciences, Speech-Language Pathology, Uppsala University, Uppsala, Sweden; 2 Department of Medical Sciences, Respiratory, Allergy and Sleep Research, Uppsala University, Uppsala, Sweden; 3 Department of Speech-Language Pathology, Uppsala University Hospital, Uppsala, Sweden; 4 Department of Women’s and Children’s Health: Physiotherapy and behavioral medicine, Uppsala University, Uppsala, Sweden; 5 Department of Physiotherapy, Uppsala University Hospital, Uppsala, Sweden; 6 Department of Medical Sciences, Clinical Physiology, Uppsala University, Uppsala, Sweden; 7 Department of Epidemiology and Global Health, Umea University, Umea, Sweden; 8 Occupational and Environmental Medicine, School of Public Health and Community Medicine, Institute of Medicine, Sahlgrenska Academy, University of Gothenburg, Gothenburg, Sweden; 9 Tartu University Hospital, Lung Clinic, Tartu, Estonia; 10 Department of Public Health, Research Unit for Environment, Occupation and Health, Danish Ramazzini Centre, Aarhus University, Aarhus, 8000 Denmark; 11 Department of Occupational Medicine, Danish Ramazzini Centre, Aarhus University Hospital, Aarhus, Denmark; 12 Department of Sleep, Landspitali University Hospital, Reykjavik, Iceland; 13 Faculty of Medicine, University of Iceland, Reykjavik, Iceland; 14 Department of Global Public Health and Primary Care, University of Bergen, Bergen, Norway

**Keywords:** Voice disorders, Voice problems, Chronic cough, Prevalence, Sick leave, General health, Pulmonary disease, Asthma

## Abstract

**Background:**

Voice problems have been reported in individuals with chronic cough, but population-based prevalence data are not yet available. While both conditions independently affect health and sick leave, their combined effects have not been studied. This study investigated the prevalence of voice problems in individuals with chronic cough and whether a cough duration of over 10 years is associated with a higher prevalence. Additionally, the relationship between chronic cough, voice problems, general health, and sick leave was explored.

**Method:**

Cross-sectional and longitudinal data from the Respiratory Health In Northern Europe, RHINE III (*n* = 7,372) and RHINE IV (*n* = 10,101) surveys were analyzed. Logistic regression was used to analyze associations between chronic cough, voice problems, general health, and sick leave.

**Results:**

Voice problems were reported by 30% of individuals with dry cough and 51% with productive cough, compared to 17% without cough. Among those with dry cough, prevalence increased from 24% (< 10 years) to 37% (> 10 years), with no significant difference for productive cough. Poor health was reported by 7.5% with dry cough and voice problems, 10% with productive cough and voice problems, and 1.7% without either condition. Chronic cough and voice problems were independently associated with poorer health, with an additive effect when co-occurring (adjusted Odds Ratio (95% CI): Dry cough 1.78 (1.34–2.37), Productive cough 2.03 (1.56–2.63), Voice problems 1.73 (1.54–1.94)). Chronic cough, but not voice problems, was linked to increased sick leave.

**Conclusion:**

Voice problems are common among individuals with chronic cough, especially in productive cough. Both chronic cough and voice problems are independently associated with poorer general health, with additive effects when co-occurring. Chronic cough, but not voice problems, was associated with more sick leave. These findings highlight the need to address the combined burden of chronic cough and voice problems to improve patient outcomes and well-being.

**Supplementary Information:**

The online version contains supplementary material available at 10.1186/s12890-025-03877-6.

## Introduction

Chronic cough affects approximately 10% of the adult population and is typically defined as a cough lasting more than eight weeks [1, 2]. It can persist for several years, with more than 40% still experiencing symptoms after five years [3]. Patients attending specialist clinics with complaints of chronic cough are predominantly women over the age of 50 [4].

In patients with chronic cough, the cough can manifest as either dry or productive (coughing up phlegm) and both types of cough can be associated with several lung diseases or can be idiopathic [1, 5]. The type of cough seems to reflect somewhat different clinical cohorts with different demographics and comorbidities [6, 7]. Chronic cough is often difficult to treat and can have a considerable impact on both mental and physical health [8–10], and has also been found to decrease work productivity and increase the number of sick leave days [6, 10, 11].

Self-reported voice problems, including symptoms such as vocal fatigue, vocal strain, and hoarseness, as well as measurable deviations in voice function have been reported in several studies on chronic cough [12–16]. In clinical studies on patients with chronic cough, up to 75% have reported voice symptoms, and dysphonia has been found in up to 40% [12, 17]. To the best of our knowledge, there are no population-based studies available on the prevalence of voice problems in individuals with chronic cough.

The cause of voice problems in chronic cough is unclear. Unexplained chronic cough is believed to result from increased sensitivity of the sensory nerves in the airways, a view that is also supported by the European Respiratory Society Task Force [18]. It has been suggested that the voice problems in individuals with chronic cough might be caused by a similar mechanism involving heightened laryngeal sensitivity [14, 19, 20]. This laryngeal hypersensitivity may contribute not only to cough, but also to increased laryngeal muscle tension and conditions such as inducible laryngeal obstruction, which in turn can lead to voice problems [14, 20]. Several factors have been proposed as potential triggers of this laryngeal sensory dysfunction, including viral infections, and gastroesophageal reflux, all of which may amplify laryngeal sensitivity and contribute to the development of both cough and voice symptoms [21].

Airway disease, including asthma, chronic rhinosinusitis (CRS), and chronic obstructive pulmonary disease (COPD) are also known risk factors for developing voice problems [22–24]. These conditions are also frequently linked to chronic cough [1, 5]. The cough itself might also result in voice problems by causing trauma to the vocal fold epithelium [25]. In long-term chronic cough, repeated vocal fold trauma might cause lesions, potentially leading to an increase in voice symptoms.

Voice problems may have a negative impact on quality of life and general health [26] and have been reported to increase the number of sick leave days [27]. However, it is unclear how general health and sick leave is affected when chronic cough and voice problems co-occur.

The main aim of this study was to examine the prevalence of voice problems in chronic cough in a general, North European population, and to determine whether a cough duration longer than 10 years is associated with a higher prevalence of voice problems compared to a shorter duration of cough. The association between voice problems in chronic cough, sick leave, and self-perceived general health was also explored.

## Method

The present study is both a cross-sectional and a longitudinal multicenter study based on data from the cohort study *“Respiratory Health in Northern Europe”* (RHINE) III and IV [28]. RHINE is a collaboration between seven Northern European centers: Aarhus (Denmark); Umea, Gothenburg, and Uppsala (Sweden); Bergen (Norway); Reykjavik (Iceland); and Tartu (Estonia). Since 1990, a random population sample (*n* = 21,802), born between 1945 and 1973, has completed questionnaires, with follow-ups every ten years. The population sample was originally derived from the participants in the European Community Respiratory Health Survey (ECRHS) [29]. Data collection for RHINE III was conducted from 2010 to 2012, and for RHINE IV from 2020 to 2023.

RHINE includes questions about respiratory symptoms and diseases, age, sex, body mass index (BMI), smoking, employment, and sick leave. In RHINE IV, a question about voice problems was also included. The questionnaire is publicly available and can be accessed online [28]. The study protocol has previously been described elsewhere [6, 30].

Data from RHINE IV were used for cross-sectional analyses, with 10,101 individuals completing the questionnaire. To examine whether a cough duration over 10 years increased the prevalence of voice problems, longitudinal data with responses from 7,372 participants who answered cough-related questions in both RHINE III and RHINE IV, as well as the question about voice problems in RHINE IV, were analyzed.

The RHINE study received approval from the Regional Committees for Medical and Health Research Ethics in compliance with national legislation. All participants gave informed consent prior to participation in each study wave.

### Chronic cough

Chronic cough was defined as a positive answer to the question *“In recent years*,* have you been troubled by a protracted cough?”* Participants who also gave a positive answer to the question *“Do you usually bring up phlegm or do you have phlegm in your lungs which you have difficulty bringing up?”* were considered to have productive cough, while the participants with a negative answer were considered to have dry cough. The same definition was used in a previous study [6]. In the present study, the term “cough” specifically refers to chronic cough. Acute or intermittent cough is not evaluated.

### Voice problems

The occurrence of voice problems was evaluated with a question previously used in a public health study examining the prevalence of voice problems in a Swedish cohort [31]. The question read “*Does your voice tire*,* strain*,* or get hoarse when you talk? Disregard symptoms that depend on current cold or upper-airway infection. The voice symptoms may vary but try to estimate an average*”, with the response options: *1 = No; 2 = Yes*,* to a small extent; 3 = Yes*,* to a great extent*. Due to the small number of participants reporting voice problems to a great extent, all individuals who reported voice problems to any extent were combined into a single category for most analyses. However, in the analyses of sick leave, participants with voice problems were also analyzed based on the extent of their symptoms.

### Chronic cough duration (< 10 years/> 10 years)

Participants with a chronic cough duration exceeding 10 years were classified based on their responses in the RHINE III and RHINE IV surveys. If they reported any cough in RHINE III and a dry cough in RHINE IV, they were categorized as having dry cough > 10 years. Conversely, if they reported any cough in RHINE III and a productive cough in RHINE IV, they were categorized as having productive cough > 10 years. Participants who reported any cough in RHINE III but no cough in RHINE IV were classified as having a chronic cough duration < 10 years.

### General health

General health was evaluated with the question *“In general*,* how would you rate your overall* health?” with the options *1 = Excellent; 2 = Very good; 3 = Good; 4 = Fair; 5 = Poor.* Responses were categorized into three groups for the unadjusted analysis: (1) Excellent and very good; (2) Good and fair; (3) Poor.

### Sick leave

The analysis of sick leave was conducted on participants currently working, defined as reporting being employed or self-employed in response to the question “*What term best describes your current work situation?”*. A total of 9,823 individuals answered the question, of whom 5,920 were currently working and therefore eligible for the analysis of sick leave. Due to missing data on cough, voice problems, and/or sick leave, the final sample size for this analysis was 5,588 participants. Sick leave was evaluated with a question about the number of sick leave days in the previous 12 months with the following response option*s: 0 = 0 days; 1 = 1–7; 2 = 8–30; 3 = 31–90; 4 = 91–365 days*. The responses were categorized into three groups for the unadjusted analysis: (1) No sick leave days; (2) 1–7 days; (3) more than 7 days.

### Confounders

Confounders were identified by using a directed acyclic graph (DAG) including variables that previous research has identified as potential confounders of the association between chronic cough (exposure) and voice problems (outcome) (See Additional file 1). The confounders identified were study center, sex, age, BMI, educational level, cardiovascular disease, asthma, CRS, COPD, nocturnal gastroesophageal reflux (nGER), smoking history, and current anxiety and/or depression. Sex and age were self-reported, and BMI was calculated from self-reported weight and height (kg/m^2^). Educational level was derived from RHINE III and participants were categorized into three levels of education: elementary school, high school and college/university. Cardiovascular disease was defined as a positive answer to the question *“Have you ever had a stroke?”* and/or the question *“Have you ever been treated in hospital because of heart infarction or angina pectoris?”* Asthma was defined as an affirmative answer to the question *“Have you had an attack of asthma in the last 12 months?”* and/or an affirmative answer to the question *“Are you currently taking any medicine (including inhalers*,* aerosols*,* or tablets) for asthma?”*, in line with definitions used in previous studies [6, 32]. CRS was defined as the occurrence of a minimum of 2 of the following symptoms for ≥ 12 weeks in the past year; nasal obstruction, pain/pressure in the upper facial region, nasal secretion, or reduced/absent smell [6]. COPD was defined as an affirmative answer to the question *“Has a doctor ever told you that you have COPD*?”. nGER was characterized as the occurrence of heartburn or belching once a week or more while in a supine position. Information about smoking history was obtained from the questions *“Are you a smoker (this applies even if you only smoke the odd cigarette/cigar or pipe every week)?”* and “*Are you an ex-smoker?”.* Participants were categorized as either never, former, or current smokers based on the answers to these questions. Current anxiety/depression was defined as an affirmative answer to one or both of these questions: “*Do you currently receive treatment for depression?”* and “*Do you currently receive treatment for anxiety?”.* Asthma, COPD, CRS, and nGER were identified as comorbidities associated with voice problems and chronic cough based on previous research. The prevalence of these conditions combined was calculated across subgroups defined by chronic cough presence and type, as well as voice problem status.

### Statistical analysis

Stata 17 (StataCorp LLC, Texas, USA) was used for statistical analyses. Descriptive statistics were used to summarize and describe the characteristics of the participants in the overall cohort as well as in the different subgroups with and without chronic cough and voice problems. The association between voice problems and chronic cough, as well as long-term chronic cough, was analyzed using the Chi-squared test. Logistic regression was performed to adjust for confounders and to conduct stratified analyses. The association of chronic cough and voice problems with general health were assessed using ordered logistic regression with the original response options on general health. For sick leave, logistic regression was performed with participants divided into two groups, those with 7 or fewer days of sick leave, and those with more than 7 days in the previous 12 months. Voice and chronic cough parameters were analyzed as separate independent variables in these regression models. Interaction terms between voice problems and cough type were included to assess whether the effect of voice problems on health and sick leave outcomes varied by the presence of cough. To evaluate the effect of sex and asthma on the outcome, the same regression models were performed, stratified by sex and asthma. The results were presented as odds ratios (OR) and 95% confidence intervals (95% CI). A p-value < 0.05 was considered statistically significant.

## Results

### Prevalence of voice problems in participants with chronic cough

Overall, 6.4% of participants reported having a chronic dry cough, and 7.5% reported a chronic productive cough (Table 1).

Among participants with dry cough, 30% reported voice problems and among those with productive cough, the prevalence was 51%. In comparison, 17% of participants without chronic cough reported voice problems, and in the total sample, 20% experienced voice problems. The difference between the groups was statistically significant (*p* < 0.0001 for all comparisons) (Table 1).

Voice problems were generally more common among women, with the highest female proportion among participants with dry chronic cough and voice problems. A higher proportion of former smokers was observed among participants with voice problems, irrespective of cough status (Table 1).

Those reporting voice problems were more likely to also report asthma compared to participants without voice problems, irrespective of cough status. COPD, CRS, nGER, and anxiety/depression were also more prevalent among those with voice problems, with the highest proportion in participants also experiencing productive cough (Table 1).

**Table 1:** Participant characteristics according to cough type and voice problems

	All	No cough *n* 8,584 [86.1%]		Dry cough *n* 636 [6.4%]		Productive cough *n* 750 [7.5%]	
		Normal voice	Voice problems	Normal voice	Voice problems	Normal voice	Voice problems
Subjects n [%]	10 101	7031 [83%]	1423 [17%]	434 [70%]	190 [30%]	359 [49%]	379 [51%]
Age (years, mean (SD))	62 ± 7	62 ± 7	62 ± 7	62 ± 7	61 ± 7	63 ± 7	63 ± 7
Female gender %	54	51	55	55	69	50	59
BMI (kg/m^2^, mean (SD))	26.5 ± 5.8	26.4 ± 5.6	26.8 ± 6.3	28.0 ± 6.5	25.2 ± 6.4	26.9 ± 6.3	25.3 ± 5.5
Smoking status %							
Never	52	54	47	56	57	44	39
Former	39	38	44	33	37	37	46
Current	8.9	7.9	8.7	11	6.4	18	15
Hypertension %	13	13	14	12	16	8.2	12
Diabetes %	7.9	7.1	8.7	12	6.8	11	12
Cardiovascular disease %	6.5	5.8	9.2	5.7	5.1	8.0	9.9
Ever-diagnosed asthma %	12	8.9	24	17	27	27	50
Current anxiety/ Depression %	7.9	6.6	10	6.9	15	11	19
**Educational level %**							
Elementary school	8.7	7.9	10	5.0	4.8	13	15
High school	40	40	41	39	39	43	39
College/university	51	52	48	56	56	44	46
Currently working %	60	61	58	64	68	53	50
Ever changed job because of breathing problems %	1.4	1.0	2.0	1.0	3.4	2.8	4.0
**Comorbidities of cough or voice problems %**							
Current asthma	12	6.6	19	15	27	30	53
COPD	2.9	1.6	4.6	3.0	4.9	10	13
CRS	6.1	3.3	9.4	6.4	15	16	31
nGER	9.4	6.9	14	11	21	16	23
Any comorbidities^#^	23	15	35	30	51	51	72

Data are presented as % or mean ± Standard Deviation (SD). BMI: Body Mass Index; COPD: Chronic Obstructive Pulmonary Disease; CRS: Chronic Rhinosinusitis; nGER: Nocturnal Gastroesophageal Reflux; #: any of current asthma, COPD, CRS and/or nGER. Data on voice problems were missing for 130 participants in the no-cough group, 12 in the dry cough group, and 12 in the productive cough group

The association between voice problems and chronic cough persisted after adjusting for confounders. Individuals with dry cough had 1.68 times the odds of experiencing voice problems compared to those without cough, the corresponding number for those with productive cough was 2.80 (Table 2).

**Table 2:** Adjusted logistic regression on the association between chronic cough and voice problems

Voice problems	Variable	OR	95% CI	*P*-value
**Total analysis (n 7,204)**				
	Dry cough	1.68	1.34–2.12	< 0.0001
	Productive cough	2.80	2.08–3.77	< 0.0001
**Stratified by sex**				
Male (n 3,416)	Dry cough	1.29	0.87–1.89	0.204
	Productive cough	2.66	1.98–3.57	< 0.0001
Female (n 3,788)	Dry cough	1.88	1.42–2.50	< 0.0001
	Productive cough	2.82	2.12–3.75	< 0.0001
**Stratified by asthma**				
Asthma (n 815)	Dry cough	1.15	0.71–1.88	0.571
	Productive cough	2.66	1.86–3.79	< 0.0001
No asthma (n 6,389)				
	Dry cough	1.91	1.48–2.47	< 0.0001
	Productive cough	2.92	2.26–3.77	< 0.0001

Three analyses were conducted: (1) total sample analysis (2) stratified by sex (male and females), and (3) stratified by current asthma (current self-reported asthma vs. no asthma). Total sample analysis was adjusted for study center, sex, age, BMI, educational level, smoking history, cardiovascular disease, current asthma, COPD, CRS, nGER and current anxiety/depression, and stratified analyses on sex and asthma were adjusted for the same confounders excluding sex and asthma respectively. Results are presented as OR; 95% CI

When stratified by sex, dry cough was significantly correlated with voice problems only in women, while productive cough showed significant correlations in both sexes. Stratification by asthma showed that productive chronic cough, but not dry chronic cough, was significantly correlated with voice problems in participants with self-reported asthma. Among those without asthma, both dry and productive chronic cough were correlated with voice problems (Table 2).

### Chronic cough duration (< 10 years/ >10 years) and voice problems

**Fig. 1:**
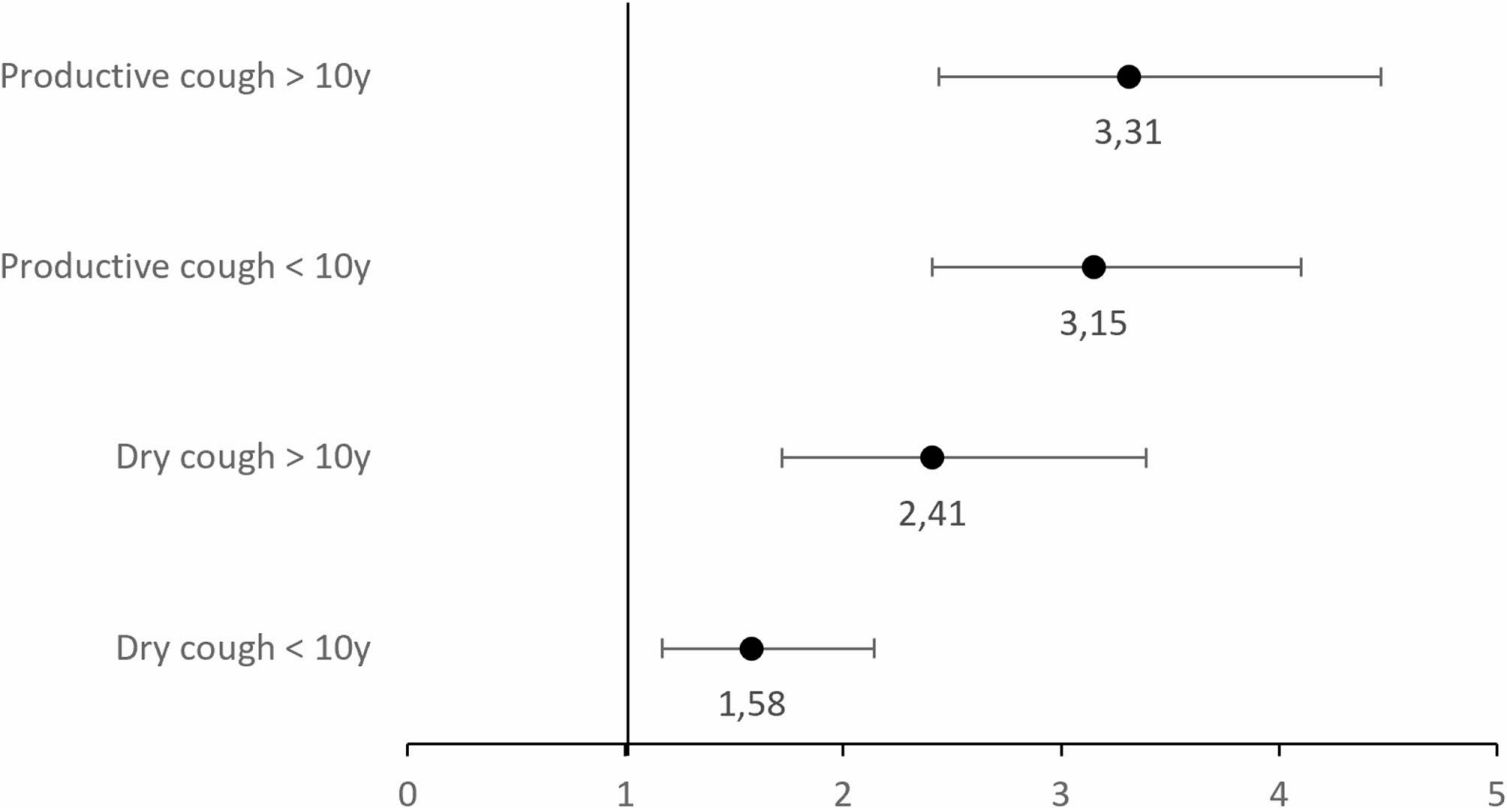
ORs and 95% CIs for voice problems by cough type and duration (< 10 or > 10 years). n = 6,492. Adjusted for study center, sex, age, BMI, educational level, smoking history, cardiovascular disease, current asthma, COPD, CRS, nGER, and anxiety/depression

Participants who reported a chronic cough duration exceeding 10 years had a higher prevalence of voice problems compared to those with a shorter duration of cough, with the biggest difference in prevalence seen in participants with dry cough. For participants with a dry cough lasting less than 10 years, 24% reported voice problems, which increased to 37% among those with a dry cough lasting more than 10 years. Among participants with productive cough, 47% of those with a cough duration under 10 years experienced voice problems, with a slight increase to 53% for those whose cough exceeded 10 years. The difference in prevalence was significant for dry cough (*p* = 0.003) but not for productive cough (*p* = 0.13). This remained largely unchanged after adjusting for confounding factors (Fig. 1).

Stratification by asthma status showed a significant correlation between productive chronic cough, but not dry chronic cough, and voice problems among participants with self-reported asthma (Table 3).

When stratified by sex, dry chronic cough was significantly correlated with voice problems in women regardless of cough duration, and in men only in those with more than 10 years of cough. Productive cough was significantly correlated with voice problems in both sexes, regardless of cough length (Table 3).

**Table 3:** Adjusted logistic regression of chronic cough duration (< 10 vs. >10 years), stratified by sex and asthma

Voice problems	Variable	OR	95% CI	*P*-value
**Stratified by sex**				
Male (n 3,115)	Dry cough < 10y	1.27	0.77–2.07	0.347
	Dry cough > 10y	2.04	1.03–3.50	0.041
	Productive cough < 10y	2.96	2.02–4.33	< 0.0001
	Productive cough > 10y	3.76	2.42–5.85	< 0.0001
Female (n 3,377)	Dry cough < 10y	1.81	1.23–2.67	0.003
	Dry cough > 10y	2.73	1.80–4.14	< 0.0001
	Productive cough < 10y	3.42	2.35–4.98	< 0.0001
	Productive cough > 10y	2.96	1.96–4.49	< 0.0001
**Stratified by asthma**				
Asthma (n 687)	Dry cough < 10y	1.10	0.56–2.17	0.790
	Dry cough > 10y	1.48	0.74–2.95	0.269
	Productive cough < 10y	3.22	1.97–5.28	< 0.0001
	Productive cough > 10y	3.14	1.96–5.04	< 0.0001
No asthma (5,805)	Dry cough < 10y	1.74	1.24–2.43	0.001
	Dry cough > 10y	2.84	1.93–4.19	< 0.0001
	Productive cough < 10y	3.07	2.23–4.24	< 0.0001
	Productive cough > 10y	3.47	2.32–5.19	< 0.0001

Logistic regression models assessing the association between cough type (dry vs. productive) and cough duration (< 10 years vs. >10 years) with the occurrence of voice problems. Two analyses were conducted: (1) stratified by sex (males and females), and (2) stratified by current asthma (current self-reported asthma vs. no asthma). Adjusted for study center, sex, age, BMI, educational level, smoking history, cardiovascular disease, current asthma, COPD, CRS, nGER, current anxiety/depression, excluding sex for analysis 1 and asthma for analysis 2. Results are presented as OR; 95% CI

### Self-rated general health

**Fig. 2:**
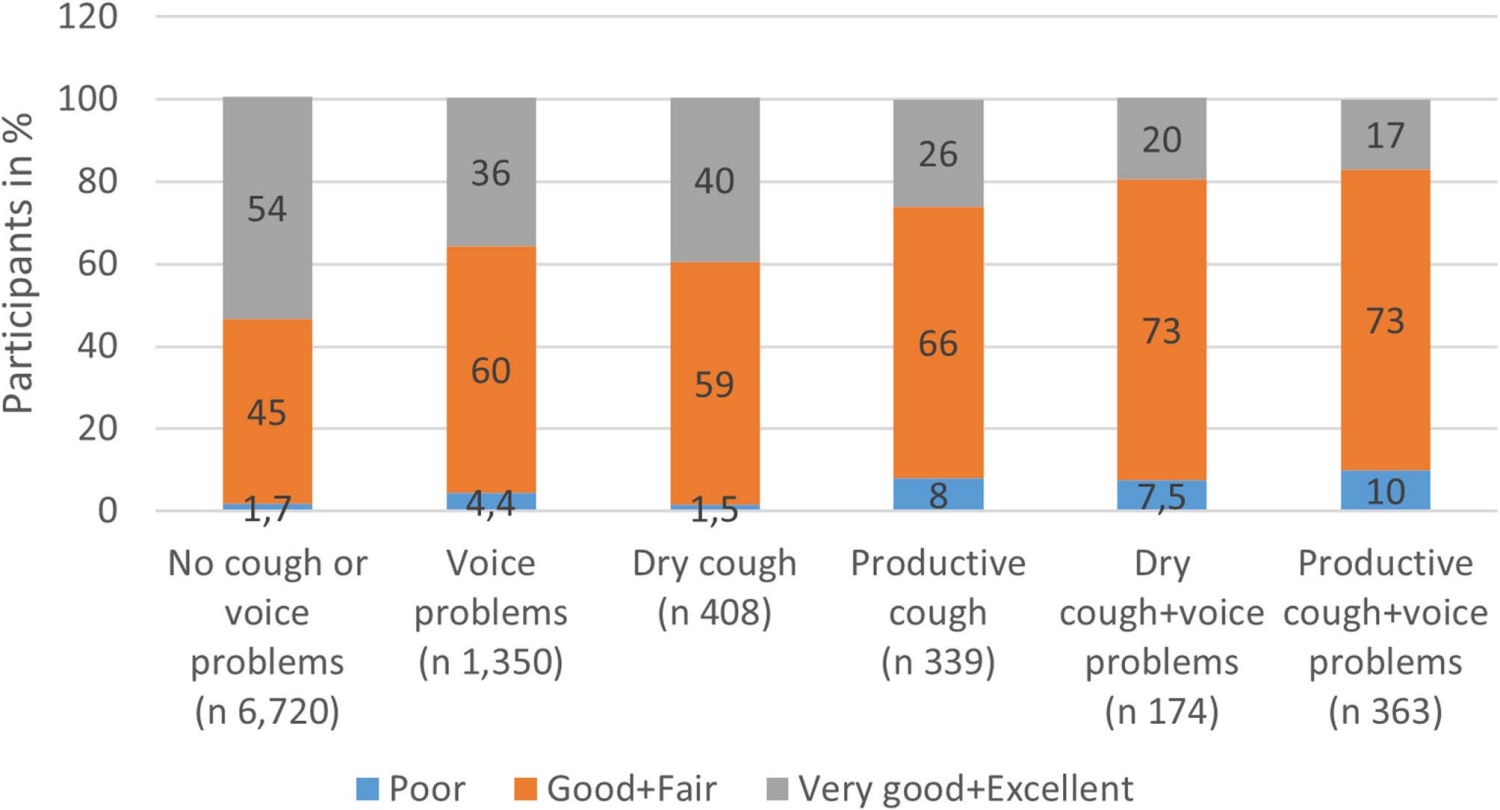
Self-Reported General Health (%) for Participants Across Different Voice and Cough Groups. The response options to the question about general health are here categorized into three groups. Percent values above ten are rounded to the nearest whole number, while values below ten are rounded to one decimal place. Percentages may not sum to 100 due to rounding

Voice problems were associated with decreased self-reported general health. Participants with voice problems and chronic cough more often reported poor health, compared to participants without both cough and voice problems (Fig. 2).

In an adjusted analysis, individuals with voice problems had 1.73-fold increased odds of reporting lower general health compared to those without voice problems. Dry cough and productive cough were also associated with poorer reported general health (dry cough: adj. OR 1.78; productive cough: adj OR 2.03). The results were consistent when stratified by sex and self-reported asthma (Table 4). An interaction analysis revealed that the effects of voice problems and chronic cough on general health were independent of each other, although the effect of voice problems was weaker among those with productive cough (*p* = 0.002).

**Table 4:** Ordered logistic regression on the association between voice problems, chronic cough, and general health

General health	Variable	OR	95% CI	*P*-value
**Total analysis** (*n* 7,120)				
	Voice problems	1.73	1.54–1.94	< 0.0001
	Dry cough	1.78	1.34–2.37	< 0.0001
	Productive cough	2.03	1.56–2.63	< 0.0001
**Stratified by sex**				
Male (n 3,388)	Voice problems	1.97	1.66–2.33	< 0.0001
	Dry cough	1.83	1.38–2.43	< 0.0001
	Productive cough	2.02	1.55–2.62	< 0.0001
Female (n 3,732)	Voice problems	1.70	1.45–1.98	< 0.0001
	Dry cough	1.56	1.24–1.97	< 0.0001
	Productive cough	2.24	1.75–2.87	< 0.0001
**Stratified by asthma**				
Asthma (n 808)	Voice problems	1.37	1.04–1.79	0.025
	Dry cough	1.77	1.15–2.73	0.010
	Productive cough	2.11	1.52–2.92	< 0.0001
No asthma (n 6,312)	Voice problems	1.82	1.61–2.07	< 0.0001
	Dry cough	1.56	1.28–1.90	< 0.0001
	Productive cough	2.20	1.77–2.74	< 0.0001

Analyses was made on the original response options for the question about general health. Three analyses were conducted: (1) total sample analysis, (2) stratified by current asthma (current self-reported asthma vs. no asthma), and (3) stratified by sex (male and females). Total sample analysis was adjusted for study center, sex, age, BMI, educational level, smoking history, cardiovascular disease, current asthma, COPD, CRS, nGER, current anxiety/depression, and stratified analyses on sex and asthma were adjusted for the same confounders excluding sex and asthma respectively. Results are presented as OR; 95% CI

### Sick leave

**Fig. 3:**
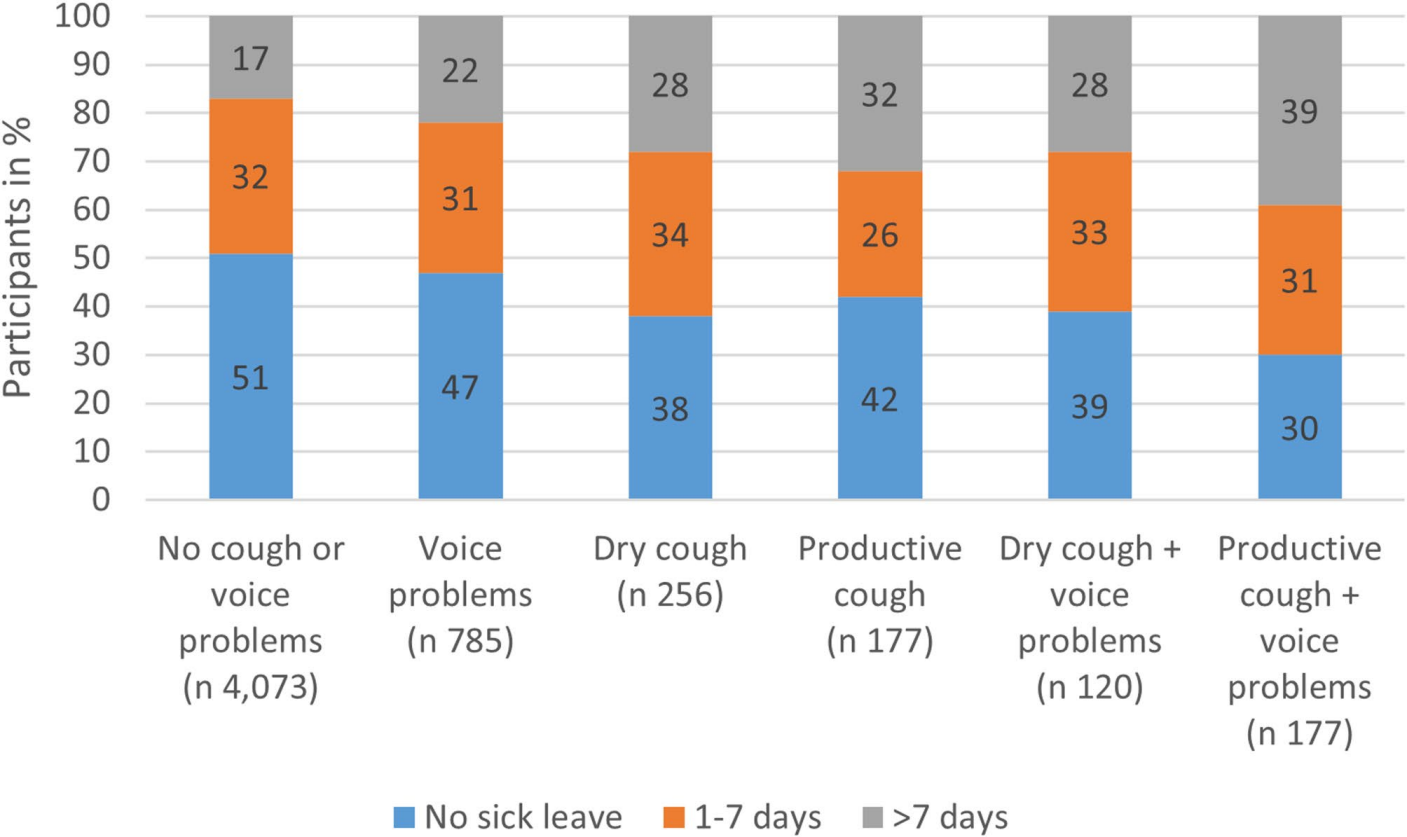
Self-reported sick leave (%) in the past 12 months by voice and cough groups. Analysis includes participants currently working with complete data on cough, voice problems, and sick leave (n = 5,588). Percent values above ten are rounded to the nearest whole number, while values below ten are rounded to one decimal place. Percentages may not sum to 100 due to rounding

A higher proportion of participants with chronic cough alone or chronic cough combined with voice problems had been on sick leave for more than 7 days during the last year compared to participants without these issues (Fig. 3).

For participants with dry cough, there was no difference in sick leave between those with and without voice problems. However, among participants with productive cough, a greater percentage of participants with both productive cough and voice problems reported more than 7 days of sick leave compared to those with productive cough alone (Fig. 3).

When adjusting for confounders, no association was found between voice problems and sick leave exceeding 7 days. When analyzing the degree of voice problems separately, the odds ratio for voice problems to a great extent was 1.34 (95% CI: 0.77–2.31, *p* = 0.296) (Table 5). Both dry cough and productive cough were significantly associated with an increase in sick leave days. An interaction analysis examining the combined effects of voice problems and chronic cough on sick leave did not reveal any significant interactions (Table 5).

**Table 5:** Adjusted logistic regression of voice problems, chronic cough, and > 7 sick leave days last year

Sick leave	Variable	OR	95% CI	*P*-value
**Total analysis** (*n* 4,258)				
	Voice problems	1.08	0.88–1.32	0.447
	Dry cough	1.62	1.22–2.16	0.001
	Productive cough	1.74	1.29–2.35	< 0.0001
**Degree of voice problems**				
	Voice problems-small extent	1.06	0.86–1.30	0.580
	Voice problems- great extent	1.34	0.77–2.31	0.296
	Dry cough	1.62	1.22–2.15	0.001
	Productive cough	1.72	1.27–2.32	< 0.0001
**Interaction analysis**				
	Voice problems# dry cough	0.68	0.37–1.26	0.215
	Voice problems# productive cough	0.93	0.52–1.66	0.799

The analyses examined the association between voice problems, chronic cough, and participants reporting more than seven days of sick leave during the previous year. Three analyses were conducted (1) the main effects of voice problems, dry cough, and productive cough on sick leave; (2) Voice problems analyzed by the degree of voice problems (small vs. great extent); and (3) interaction analyses between voice problems and both types of cough (dry and productive). All analyses were adjusted for study center, sex, age, BMI, educational level, smoking history, cardiovascular disease, current asthma, COPD, CRS, nGER, and current anxiety/depression. Results are presented as OR; 95% CI. Analyses were performed on participants currently working

## Discussion

In this population-based study of individuals aged 48–76 years, a high prevalence of self-reported voice problems among those with chronic cough was found: Approximately one-third of participants with dry chronic cough and half of those with productive chronic cough reported voice problems, compared to less than one-fifth of those without cough. Participants with dry chronic cough lasting over 10 years were significantly more likely to report voice problems compared to those with a shorter duration of cough. These associations remained also after adjusting for confounding factors.

Chronic cough and voice problems were independently associated with reported poorer general health, a pattern that persisted after adjusting for confounding factors and was not influenced by comorbid asthma or sex. There was, however, no clear association between self-reported voice problems and an increased number of sick leave days.

### Prevalence of voice problems in participants with chronic cough

To the best of our knowledge, the current study is the first population-based study examining the prevalence of self-reported voice problems among individuals with chronic cough. Previous studies on voice problems in chronic cough have primarily focused on clinical cohorts, reporting prevalence rates between 40% and 75%, without differentiating between dry or productive chronic cough [12, 13, 16, 17]. In the present study, a higher prevalence of voice problems was observed among individuals with productive chronic cough, with approximately half experiencing voice problems, compared to about 30% of those with dry cough. This difference suggests that distinct underlying mechanisms might contribute to voice problems in these groups. For instance, smoking history and respiratory-related comorbidities, such as COPD, asthma and CRS appeared more common in individuals with productive chronic cough and voice problems, indicating that differences in airway function could influence the likelihood of developing voice problems. Although studies comparing individuals with dry and productive chronic cough have shown mixed results regarding differences in demographics, symptoms, and comorbidities [6, 7, 33, 34], the present study suggests that differences in comorbidities and frequency of voice problems may distinguish these groups.

In the current study, 17% of participants without cough reported voice problems, while the prevalence in the total cohort was 20%. In the general adult population, the prevalence of self-reported voice problems varies, from approximately 6–40%, depending on definition, study method, and demographic factors [27, 31, 35, 36]. A Swedish study (n 74,351), which used the same question regarding voice problems as in the current study, reported a prevalence of approximately 17% among adults aged 18–104 years [31]. In the present study, participants ranged in age from their late forties to late seventies, which may account for the slightly higher prevalence observed, as voice problems are more common in older adults [31, 37].

Among participants with voice problems, self-reported asthma was more common compared to those without voice problems, with the highest proportion observed in individuals with both productive chronic cough and voice problems. This suggests that asthma is an important contributing factor to the development of voice problems, regardless of the presence of chronic cough. Asthma is a known risk factor for developing voice problems, likely due to a combination of airway inflammation, bronchoconstriction, and the effects of inhaled corticosteroids commonly used in asthma management [24, 38]. Inducible laryngeal obstruction (ILO), a condition that can mimic or coexist with asthma (and is sometimes misdiagnosed as asthma), may also contribute to voice problems [39]. These overlapping mechanisms may help explain the observed loss of significant association between dry chronic cough and voice problems, when stratifying by asthma. This suggests that asthma-related factors, such as airway changes, treatment side-effects, or coexisting laryngeal dysfunction, may have a more pronounced influence on vocal health than dry chronic cough alone.

In the present study, dry chronic cough lasting longer than 10 years was associated with higher odds of voice problems compared to a shorter duration of cough. A potential explanation for this finding could be the repeated trauma to the vocal folds caused by prolonged coughing, which may lead to vocal fold edema, irritation or the development of lesions [25]. However, a study by Adessa, et al. [40] found no difference in the prevalence of benign vocal fold lesions between individuals with longer or shorter cough durations, suggesting that additional mechanisms beyond vocal fold lesions may be responsible. One possibility is that prolonged coughing maintains or exacerbates an underlying laryngeal hypersensitivity, which over time may contribute to increased laryngeal muscle tension and the development of voice problems. Laryngeal hypersensitivity has been proposed as an underlying mechanism for both voice problems and chronic cough itself [14, 19, 20].

Compared to dry chronic cough, the odds of experiencing voice problems in participants with productive cough did not increase with a cough duration over 10 years. The reason for this difference remains unclear. However, individuals with productive chronic cough were found to have higher odds of voice problems compared to those with dry cough regardless of cough duration. Factors such as smoking and conditions like COPD, chronic bronchitis, and bronchiectasis, more often linked to productive chronic cough, may contribute to this increased susceptibility by affecting both laryngeal structures and respiratory function [23, 41]. In the present study, comorbidities such as COPD, asthma, CRS, and nGER were more frequently reported among participants with productive chronic cough and voice problems. These conditions may have contributed to the elevated odds of voice problems in this group. However, since productive cough remained associated with voice problems also after adjusting for these conditions, it appears to have an independent effect on voice problems.

### Self-rated general health and sick leave

Both voice problems and chronic cough are independently known to impact various health domains [8, 26]. In the current study, voice problems and chronic cough were independently associated with decreased general health, regardless of all identified confounding factors. Moreover, the combination of voice problems and chronic cough had an additive effect on decreased general health, with the lowest general health reported by the group with voice problems and productive chronic cough. While the interaction analysis indicated that the association between voice problems and general health was slightly weaker in those with productive cough, this association remained significant. In a clinical study including 430 participants, DeVore, et al. [42] also found negative effects of self-reported voice problems on general health, using a similar question as in the current study. Additionally, studies on chronic cough have confirmed the health burden associated with the condition [8, 9]. The fact that the co-occurrence of chronic cough and voice problems has a stronger association with decreased general health underscores the importance for healthcare providers involved in chronic cough care to recognize and address symptoms of voice problems in these patients. Additionally, there is a critical need to develop treatment strategies that can positively impact both chronic cough and voice problems.

The present study found no association between self-reported voice problems and sick leave exceeding 7 days, whereas an association was observed for both chronic cough groups, consistent with previous studies [6, 10]. The lack of association for voice problems is somewhat unexpected, given earlier findings of increased sick leave among individuals with voice problems, particularly in voice-demanding occupations (such as teachers, early childhood educators, and telemarketers) [27, 43–45]. However, occupational data were not available in this study, which may have limited the ability to detect such effects.

Previous studies have often assessed voice-related sick leave specifically [27, 43, 45], while the present study used a general question about sick leave for any reason. This broader approach may have diluted any potential association. Moreover, earlier research has shown that more severe voice problems are associated with an increased number of sick leave days [45]. In this study, the majority of participants reported mild voice problems, and only a small proportion reported voice problems to a great extent. The small size of this subgroup may have limited the ability to detect an association. Although the estimates suggested somewhat higher odds of sick leave among those with voice problems to a great extent, the association was not statistically significant. Mild voice problems may not lead to extended absence, particularly in less voice-demanding occupations, and may be managed through brief voice rest or workplace accommodations. In the present study, participants with voice problems were more likely to report having changed jobs due to breathing problems potentially reflecting a shift to a less vocally demanding occupation, thereby reducing the need for sick leave.

The fact that the present study found an association between chronic cough and an increase in sick leave days that were not found in participants with voice problems could be due to several factors. For example, sleep disturbances, decreased physical ability, depression, pain, and fatigue might be more common in individuals with chronic cough than with voice problems, leading to an increased risk of sick leave in this group [8–10].

### Strengths and weaknesses

A key strength of the current study is its multicenter design, including seven study centers from Northern Europe. This design, along with a large general population cohort, enhances the relevance of the findings to the population within the included age group in Northern Europe. Another strength is that voice problems were evaluated with a validated question that has previously been used to assess the prevalence of voice problems in a Swedish cohort [31].

Some methodological limitations need to be addressed. In the present study, the definition of chronic cough did not correspond to the typical clinical definition of a cough lasting longer than 8 weeks. This lack of a specific time frame may have led to classification errors affecting the prevalence of chronic cough. However, the definition used here has been shown to identify a similar or slightly higher prevalence of chronic cough as previous studies [6]. Additionally, since long-term chronic cough was defined as self-reported chronic cough at two discrete time points, it cannot be determined whether participants experienced symptoms consistently throughout the interval, which might have influenced the observed association between long-term cough and voice problems.

In this study, participants who reported voice problems to a small extent and to a great extent were combined into a single category of “voice problems”. This was done due to the low number of participants reporting problems to a great extent, leading to significantly reduced statistical power if analyzed separately. However, previous studies have shown that even mild voice problems can impact social, physical and, mental health [42], making this combined group relevant to study.

Given the lack of longitudinal data on voice problems in this study, it is not possible to conclude causality between chronic cough and voice problems. In a retrospective chart study on 105 patients with chronic cough and hoarseness, 86% reported voice problems after the diagnosis of chronic cough, suggesting a potential temporal relationship [46]. However, these results also do not establish causality, and further research is needed to clarify the underlying mechanisms and temporal patterns.

General health was assessed using a single, global question rather than using a full questionnaire. This approach, often used for assessing self-rated health, is comparable to multi-item health assessments [47]. Additionally, the number of sick leave days during the last year was based on self-reported data and not verified by any register data, potentially introducing recall bias.

The definitions of confounders such as asthma and nGER, based on self-reported questionnaire items, may be subject to misclassification due to recall bias or lack of clinical verification. Moreover, the questionnaire assessed nGER only, not reflux more broadly. However, these definitions have been used in several previous population-based studies and shown acceptable validity [32, 48].

Loss to follow-up from the initial RHINE cohort may have led to nonresponse bias. However, a previous study examining the implications of loss to follow-up in RHINE found minimal differences between long-term responders and baseline participants indicating that nonresponse bias is unlikely [30].

## Conclusion

Voice problems are common among individuals with chronic cough in this North-European cohort, being most prevalent in individuals with productive cough. Long-term cough was more strongly associated with voice problems compared to shorter cough durations, especially in cases of dry cough. These findings highlight the importance of early management of chronic cough to prevent or alleviate the severity of voice problems. Both voice problems and chronic cough were associated with decreased general health and, when these conditions co-occur, their impact is additive. This emphasizes the importance for healthcare providers to identify and address both conditions at an early stage. Although chronic cough was found to increase sick leave, the same association was not found for voice problems.

Further longitudinal studies are needed to better understand the relationship between chronic cough and voice problems. Additionally, research focusing on the association between chronic cough, voice problems, and sick leave across various occupational groups is needed to identify at-risk populations. Moreover, interventional studies are needed to assess the effect of targeted treatment strategies for chronic cough and voice problems on quality of life, general health and sick-leave.

## Supplementary Information

Supplementary Material 1

## Data Availability

The datasets analyzed during the current study are not publicly available due to regulatory demands but are available from the corresponding author on reasonable request.

## Abbreviations

BMI: Body mass index
CI: Confidence interval
COPD: Chronic obstructive pulmonary disease
CRS: Chronic rhinosinusitis
DAG: Directed acyclic graph
ECRHS: European Community Respiratory Health Survey
nGER: Nocturnal gastroesophageal reflux
OR: Odds ratio
RHINE: Respiratory Health in Northern Europe
SD: Standard deviation
