# Supplementary figures and images for: Voice problems in chronic cough: prevalence and implications for health and sick leave in a Northern European population
Source: BMC Pulm Med. 2025 Aug 21;25:402. doi: 10.1186/s12890-025-03877-6 (PMC12369044; doi:10.1186/s12890-025-03877-6)

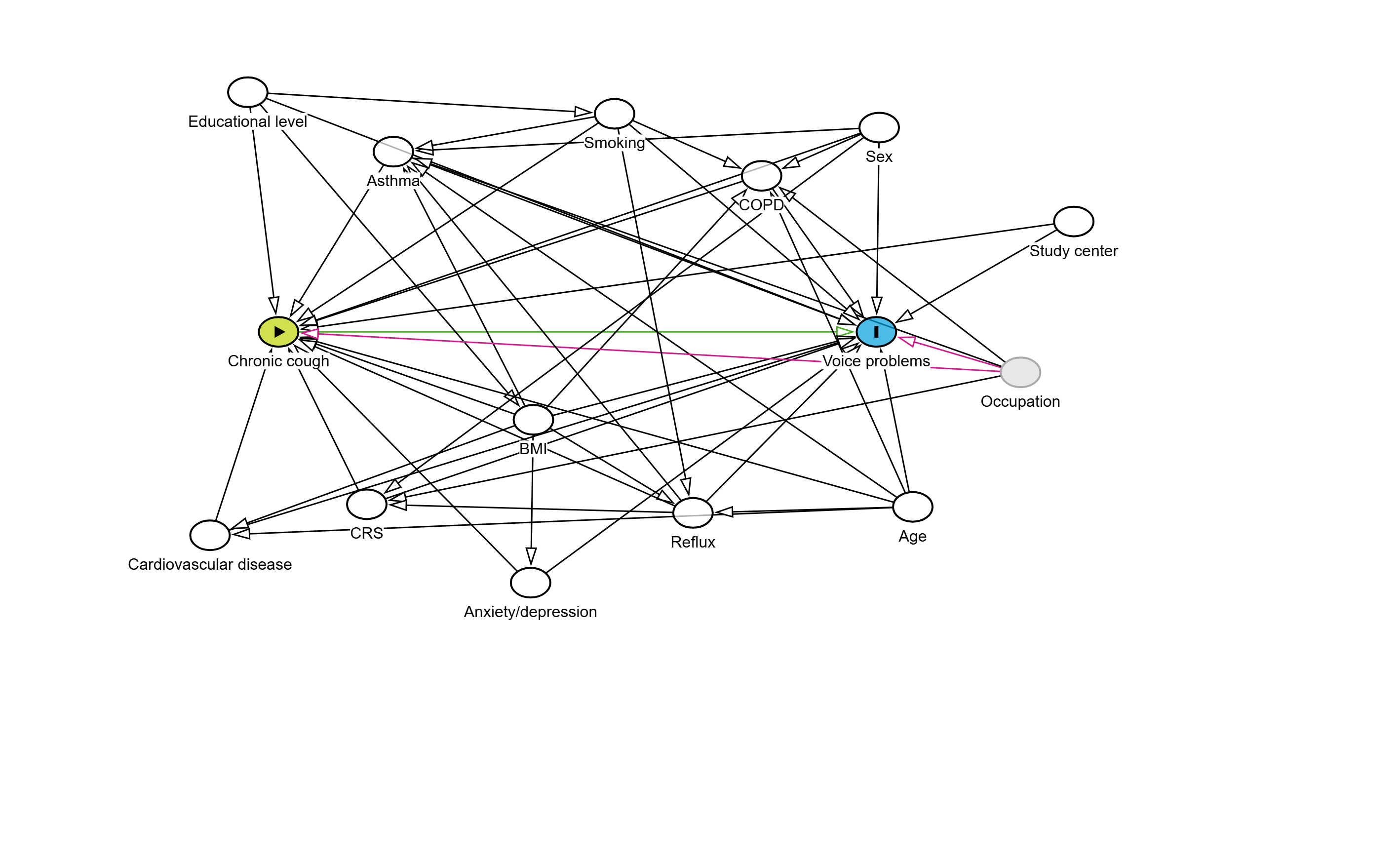

Supplement: Supplementary file 1 — Supplementary Material 1 [file 12890_2025_3877_MOESM1_ESM.png]
